# Long-Term Educational Outcomes of Individuals Born Preterm

**DOI:** 10.1001/jamanetworkopen.2025.34918

**Published:** 2025-10-01

**Authors:** Tianna Loose, Ophelie Collet, Anne Monique Nuyt, Jean-Christophe Goulet-Pelletier, Frank C. Worrell, Sylvana Côté, Thuy Mai Luu

**Affiliations:** 1 Department of Social and Preventive Medicine, School of Public Health, Université de Montréal, and CHU Sainte Justine Azrieli Research Center, Montreal, Quebec, Canada; 2 CHU Sainte Justine Azrieli Research Center and Department of Pediatrics, Faculty of Medicine, Université de Montréal, Montreal, Quebec, Canada; 3 Berkeley School of Education, University of California, Berkeley

## Abstract

**Importance:**

Children born preterm may face neurodevelopmental challenges. Data on how this translates into long-term educational outcomes at the population level are scarce.

**Objective:**

To quantify relative contributions of preterm birth and sociodemographic factors to educational achievement and attainment.

**Design, Setting, and Participants:**

This case-control study included a birth cohort of all infants born preterm in Quebec, Canada, between 1976 and 1995, matched 1:2 with infants born term. Births before 37 weeks’ gestations were considered preterm. The control cohort born between 37 and 42 weeks’ gestation were matched by birth year, sex, and pregnancy type (singleton, twins). Administrative database linkages provided objective data for longitudinal follow-up to age 43 years. Individuals who died in 1976 to 2019 or without Quebec Ministry of Education records were excluded. Analyses were performed from February 2, 2023, to June 4, 2025.

**Exposure:**

Preterm birth, defined as extremely preterm (under 28 weeks), very preterm (28 to <32 weeks), or moderate-to-late preterm (32 to <37 weeks).

**Main Outcomes and Measures:**

Outcomes included high school performance (final high school average), high school graduation by age 22 years, and university degree at any age. Sociodemographic factors were extracted from the Quebec Births and Deaths registry. Socioeconomic status was derived using postal code and the Material and Social Deprivation Index.

**Results:**

Of 297 820 participants (160 980 male [54.0%]; 28 040 individuals [9.4%] born to mothers born outside Canada), 1915 (0.6%) were born extremely preterm, 13 225 (4.4%) very preterm, 83 105 (27.9%) moderate-to-late preterm, and 199 575 (67.0%) full term. Associations of preterm birth and final high school average were negligible among those who remained in school. Gestational age was associated with educational attainment in a dose-response pattern wherein individuals born extremely preterm were the most at risk for not graduating high school (OR, 1.80; 99% CI, 1.54-2.09) or not obtaining a university degree (OR, 1.68; 99% CI, 1.39-2.02). The contribution of preterm birth was small compared with male sex, maternal educational level, socioeconomic status, and migration related factors.

**Conclusions and Relevance:**

In this case-control study, individuals born preterm were more likely than full-term counterparts to not complete high school, yet sociodemographic factors were more important than preterm status for educational attainment. Our findings substantiate the need for long-term follow-up and support to children born preterm and their families.

## Key Points

**Question:**

Do children born preterm face educational challenges into adulthood?

**Findings:**

In this case-control study including all individuals born preterm in Quebec, Canada, between 1976 and 1995, matched 1:2 with peers born full-term (297 820 individuals total), individuals born preterm were at increased odds of not graduating high school or obtaining a university degree in a dose-response pattern by gestational age at birth. However, high school grades were similar among those who remained in school, and the contribution of preterm birth was minor compared with other sociodemographic factors.

**Meaning:**

These results suggest that continuing educational support is warranted to optimize school achievement for individuals born preterm.

## Teaser

This case-control study of all births in a Canadian province examines the association of preterm birth with educational outcomes.

## Introduction

The incidence of preterm birth (earlier than 37 weeks’ gestation) has risen to about 10% of all births.^1^ In high-income countries, most infants born preterm now survive.^2^ With preterm birth, organ systems are still developing. Early exposure to noxious factors (eg, inflammation, hypoxia, pain) can affect optimal brain growth and maturation potentially leading to challenges with neurodevelopment,^3^ mental well-being,^4,5^ and academic achievement and attainment.^6,7^ Although early life support and additional resources such as physical, occupational and/or speech therapy, and extra tutoring at school for learning disabilities can mitigate vulnerabilities, socioeconomic barriers to additional services may exacerbate challenges among children born preterm.^8^

Few researchers have conducted studies on long-term educational outcomes across the full spectrum of preterm birth using large population-based cohorts that account for other health-related determinants and socioeconomic factors. Although birth at earlier gestational ages is demonstrably associated with greater learning difficulties in a dose-response pattern,^7,9,10,11,12,13^ it remains unclear whether these educational setbacks persist into adolescence and adulthood. Findings from some studies suggest potential catch-up,^14,15^ while other results indicate disadvantages lasting into adulthood in terms of education, employment opportunities, and income.^6,7,16,17,18^

Existing research on preterm birth and educational outcomes has other limitations. For instance, due to the approximately 10% incidence of preterm birth in the general population, population-based cohort studies include relatively few individuals born preterm, and even fewer born extremely preterm.^11^ Other studies cover only a partial spectrum of preterm birth,^19^ dichotomize preterm vs term status,^20^ lack control groups,^7^ or have small sample sizes.^21^ Many studies do not include the role of socioeconomic status (SES) in shaping educational outcomes, potentially missing the compounded disadvantage.^7^

To address these gaps, we designed a birth cohort case-control study using all infants born preterm in Quebec, Canada, between 1976 and 1995 matched 1:2 with infants born full-term. We examined how varying degrees of prematurity were associated with long-term educational outcomes, specifically high school grades, high school graduation rates, and postsecondary studies. We accounted for a range of sociodemographic factors to quantify the relative importance of preterm birth and explored to what extent socioeconomic factors influenced educational challenges.

## Methods

### Participants

In this birth cohort case-control study, we examined all live preterm births (23 to <37 weeks of gestation, cases) in Quebec, Canada, from January 1, 1976, to December 31, 1995, as recorded in the Quebec Births and Deaths Registry (Registre des événements démographiques [RED]). For each preterm birth, 2 individuals born full term (37 to <42 weeks’ gestation, controls) were selected from the same registry, matched by year of birth, sex, and pregnancy type (singleton, twin). Multiple pregnancies or births with triplets or more were excluded, as were participants who died in the period of 1976 to 2019 (recorded death certificates) or who did not have Quebec Ministry of Education records.

We obtained approval from our institutional Research Ethics Board and the Quebec Institute of Statistics (Institut de la Statistique du Quebec [ISQ]). We used a data management hub (Digital Matrix Systems) to guarantee data confidentiality, as it enables temporary data storage and access by authorized researchers only for the duration of the study. The ISQ limits access to anonymized data only. We followed the Strengthening the Reporting of Observational Studies in Epidemiology (STROBE) reporting guideline.

### Measures

#### Exposure and Outcomes

With previously confirmed validity of gestational age in the RED database,^22^ we categorized births as: extremely preterm (<28 weeks), very preterm (28 to <32 weeks), moderate-to-late preterm (32 to <37 weeks), and term (37 to <42 weeks).

Administrative database linkages provided objective, unbiased data for longitudinal educational follow-up to age 43 years. In Quebec, students typically graduate high school at ages 16 to 17 years, earning a high school leaving certificate known as Quebec Secondary School Diploma (Diplôme d’études secondaires [DES]). Following this, students attend CÉGEP (Collège d’enseignement général et professionnel), a unique form of postsecondary school exclusive to Quebec, for 2 to 3 years. After completion, youth can enter the university at approximately age 19 to 20 years. For this study, high school graduation was defined as obtaining the DES by age 22 years. A university degree at any age was counted.

For each participant, we measured high school performance using the final high school average as recorded in the Quebec Ministry of Education database. The final high school average takes into account all marks obtained in grades 10 and 11, the last 2 years of high school (also known as Secondary IV and V). Marks and averages are expressed as percentages. Pass or fail cut-off for each subject is 60%. If a student failed and repeated a grade, the initial and subsequent scores are both included in the average.^23^

#### Covariates

We extracted from the RED database all sociodemographic factors with less than 3% missing data, that were associated with both exposure and outcome variables. Birth characteristics included: year of birth, birth order (first-born [yes or no]), and sex (male; female) and history of stillbirths (none; 1 or more). Maternal and neighborhood covariates included mother tongue (English or French; other), language spoken at home at birth of child (French or English; other), birthplace (Canada; outside Canada), matrimonial status at birth of child (married; not married), maternal education (no high school diploma; high school diploma), and neighborhood socioeconomic status (lowest quintile; other quintiles) (eMethods in Supplement 1).

### Statistical Analysis

We used linear regression (B coefficients) to test for associations between preterm status and final high school average. We used logistic regression (odds ratios [ORs] with 99% CIs) for preterm status and high school graduation or university degree. The group born term was the reference in all models. All analyses were 2-tailed and used 99% CIs to reduce type I errors due to the large sample size.^24^ Regression analyses were conducted adjusting only for matching variables (birth year, pregnancy type, sex) and additionally including other factors. Forest plots were provided to showcase regressions.

We excluded any participants with missing data in outcomes. All participant counts were rounded to the nearest multiple of 5 to comply with ethics. We conducted analyses using SAS version 8.3 (SAS Institute Inc) and SPSS Statistics version 22 (IBM Corp). Analyses were performed from February 2, 2023, to June 4, 2025.

## Results

### Participants

Of 320 870 eligible births, we excluded 17 870 individuals without Quebec Ministry of Education records, as well as 5185 who died between 1976 and 2019, leaving 297 820 final participants (160 980 male [54.0%]; 28 040 individuals [9.4%] born to mothers born outside Canada); 1915 individuals (0.6%) were born extremely preterm (mean [SD] birthweight, 1.16 [0.98] kg), 13 225 (4.4%) very preterm (1.78 [0.75] kg), 83 105 (27.9%) moderate-to-late preterm (2.56 [0.60] kg), and 199 575 (67.0%) full term (3.33 [0.58] kg). Maternal education levels differed by preterm status, with 20.6% (395 of 1915 births), 24.0% (3180 of 13 225 births), 23.0% (19 135 of 83 105 births), and 20.3% (40 565 of 199 575 births), respectively, reporting fewer than 11 years of schooling (Table 1). Preterm birth groups had slightly higher frequencies than controls of mothers born outside Canada, not officially married, or with previous stillbirths. Similarly, somewhat higher percentages of preterm groups reported main language at home other than French or English, being first-born, or low neighborhood SES (eg, 350 of 1915 [18.3%] of extremely preterm births vs 28 500 of 199 575 [14.3%] of controls were in lowest quintile). The extremely preterm group also had more recent birth years (eg, 700 of 1915 [36.6%] were born between 1991 and 1995 vs 48 610 of 199 575 [16.7%] between 1976 and 1980), likely reflecting higher survival rates (Table 1). There was an overall increase in preterm births in Quebec, from 22 265 of 488 881 births (4.6%) for 1976 to 1980 to 28 335 of 463 399 births (6.1%) for 1991 to 1995 (eTable 1 in Supplement 1). Missing data for high school diploma attainment and final average grades in high school according to prematurity status are detailed in eTable 1 in Supplement 1. A higher proportion of missing data for final average grades was observed with decreasing gestational age, indicating an inverse association between gestational age and data completeness for this outcome.

**Table 1.** Characteristics of Term and Preterm Birth Cohorts, 1976-1995

Participants	Births, No. (%)^a^			
	Full term (37 to <42 wk) (n = 199 575)	Moderate-to-late preterm (32 to <37 wk) (n = 83 105)	Very preterm (28 to <32 wk) (n = 13 225)	Extremely preterm (<28 wk) (n = 1915)
**Birth characteristics**				
Birth year				
1976-1980	48 610 (24.4)	18 865 (22.7)	3080 (23.2)	320 (16.7)
1981-1985	46 715 (23.4)	19 005 (22.9)	3220 (24.3)	380 (19.8)
1986-1990	49 635 (24.9)	21 170 (25.5)	3350 (25.3)	520 (27.2)
1991-1995	54 610 (27.4)	24 065 (29.0)	3570 (27.0)	700 (36.6)
Birth weight, mean (SD), kg	3.33 (0.58)	2.56 (0.60)	1.78 (0.75)	1.16 (0.98)
Not first-born child	111 860 (56.0)	42 695 (51.4)	6670 (50.4)	945 (49.3)
Sex				
Female	91 540 (45.9)	38 350 (46.1)	6015 (45.5)	935 (48.8)
Male	108 035 (54.1)	44 755 (53.9)	7210 (54.5)	980 (51.2)
Previous stillbirths (≥1)	9215 (4.6)	4980 (6.0)	1060 (8.0)	170 (8.0)
**Maternal and neighborhood characteristics**				
Age at childbirth, mean (SD), y	27.2 (4.7)	27.1 (5.0)	27.0 (5.2)	27.4 (5.4)
Primary language not French or English	15 195 (7.6)	5965 (7.2)	1005 (7.6)	185 (9.7)
Language spoken at home neither French nor English	9475 (4.7)	3785 (4.6)	675 (5.1)	110 (5.7)
Born outside Canada	18 530 (9.3)	7755 (9.3)	1450 (11.0)	305 (15.9)
Not married^b^	51 030 (25.6)	25 860 (31.1)	4380 (33.1)	705 (36.8)
Education level <11 y of schooling^c^	40 565 (20.3)	19 135 (23.0)	3180 (24.0)	395 (20.6)
Neighborhood SES at age 12 y, lowest quintile^d^	28 500 (14.3)	13 505 (16.3)	2405 (18.2)	350 (18.3)

Abbreviation: SES, socioeconomic status.

^a^ All characteristics shown differ significantly by preterm status (*P* < .001), except for sex (*P* = .03).

^b^ In Quebec, many couples live in common-law relationships, without formal marriage.

^c^ Not including kindergarten, ie, likely did not finish high school (grade 11 in Quebec).

^d^ SES, socioeconomic index, as defined by postal code and the Material and Social Deprivation Index from the Quebec Public Health Institute (Institut national de santé publique du Québec [INSPQ]).

### Primary Outcome

There were no meaningful differences in high school performance (academic achievement) by group, as measured by mean (SD) final high school average: 69.4 (10.7) for extremely preterm, 70.2 (10.8) very preterm, 70.7 (10.9) moderately preterm, and 71.0 (10.8) term. However, participants born preterm showed considerably lower levels of high school graduation, in a dose-response pattern: 770 (40.2%) of those born extremely preterm did not graduate high school, 4545 (34.4%) very preterm, 25 820 (31.1%) moderately preterm, and 54 120 (27.1%) born full term. Correspondingly, 1595 (83.3%), 10 610 (80.2%), 64 965 (78.2%), and 151 360 (75.8%), respectively, did not obtain university degrees.

### Regression Analyses

Linear regression analyses showed that associations between preterm status and final high school average were significant but negligible, with a B coefficient range of 0.15 to 1.45 (Table 2; Figure). The greatest associations with low average marks were low maternal education (B = 4.43 [99% CI, 4.27-4.59]), male sex (B = 2.84 [99% CI, 2.73-2.96]), low neighborhood SES (B = 2.30 [99% CI, 2.17-2.43]), not being first-born (B = 2.30 [99% CI, 2.17-2.43]) and mother not married (B = 1.98 [99% CI, 1.83-2.14]).

**Table 2.** Associations Between Preterm Status and Educational Outcomes, Using Term Birth Group as Reference

Characteristic	Final high school average, linear regression models, B (99% CI)		OR (99% CI)			
			No high school diploma, logistic regression models		No university diploma, logistic regression models	
	Minimal adjustment	Full adjustment	Minimal adjustment	Full adjustment	Minimal adjustment	Full adjustment
Exposure						
Moderate-to-late preterm	0.31 (0.18 to 0.44)	0.15 (0.01 to 0.28)	1.22 (1.20 to 1.25)	1.16 (1.12 to 1.19)	1.15 (1.12 to 1.18)	1.10 (1.07 to 1.14)
Very preterm	0.86 (0.57 to 1.15)	0.59 (0.28 to 0.89)	1.43 (1.36 to 1.51)	1.34 (1.26 to 1.42)	1.31 (1.23 to 1.39)	1.24 (1.16 to 1.33)
Extremely preterm	1.77 (0.98 to 2.57)	1.45 (0.60 to 2.30)	1.88 (1.66 to 2.12)	1.80 (1.54 to 2.09)	1.64 (1.40 to 1.92)	1.68 (1.39 to 2.02)
Birth characteristics						
Child birth year	0.04 (0.03 to 0.05)	0.01 (0.00 to 0.02)	1.01 (1.01 to 1.01)	1.02 (1.02 to 1.02)	1.00 (1.00 to 1.00)	1.02 (1.01 to 1.02)
Twin birth	–0.15 (–0.34 to 0.03)	0.39 (0.20 to 0.58)	1.11 (1.07 to 1.15)	1.14 (1.10 to 1.19)	1.09 (1.05 to 1.13)	1.13 (1.09 to 1.18)
Male infant	2.66 (2.54 to 2.77)	2.84 (2.73 to 2.96)	2.24 (2.19 to 2.29)	2.65 (2.58 to 2.72)	2.01 (1.96 to 2.06)	2.16 (2.11 to 2.22)
Not first-born	NA	2.30 (2.17 to 2.43)	NA	1.78 (1.73 to 1.83)	NA	1.69 (1.64 to 1.74)
≥1 Previous stillbirths	NA	0.43 (0.17 to 0.70)	NA	1.19 (1.13 to 1.25)	NA	1.09 (1.03 to 1.16)
Maternal and neighborhood characteristics						
Maternal age at child birth	NA	0.35 (0.34 to 0.37)	NA	1.08 (1.08 to 1.08)	NA	1.10 (1.09 to 1.11)
Maternal mother tongue neither French nor English	NA	0.17 (0.22 to 0.56)	NA	1.17 (1.06 to 1.28)	NA	1.37 (1.27 to 1.49)
Language spoken at home neither French nor English	NA	0.38 (0.06 to 0.83)	NA	1.28 (1.16 to 1.42)	NA	1.37 (1.27 to 1.49)
Mother born outside Canada	NA	0.20 (0.08 to 0.48)	NA	1.24 (1.17 to 1.32)	NA	1.09 (1.03 to 1.15)
Mother not married^a^	NA	1.98 (1.83 to 2.14)	NA	1.88 (1.82 to 1.93)	NA	1.61 (1.55 to 1.66)
Maternal education level <11 y of schooling^b^	NA	4.43 (4.27 to 4.59)	NA	2.92 (2.83 to 3.01)	NA	3.52 (3.37 to 3.67)
Neighborhood SES (lowest quintile)^c^	NA	1.87 (1.69 to 2.04)	NA	1.63 (1.58 to 1.68)	NA	1.70 (1.63 to 1.77)

Abbreviations: NA, not applicable; OR, odds ratio; SES, socioeconomic status.

^a^ In Quebec, many couples live in common-law relationships, without formal marriage.

^b^ Not including kindergarten, ie, likely did not finish high school (grade 11 in Quebec).

^c^ SES, socioeconomic index, as defined by postal code and the Material and Social Deprivation Index from the Quebec Public Health Institute (Institut national de santé publique du Québec [INSPQ]).

**Figure.**
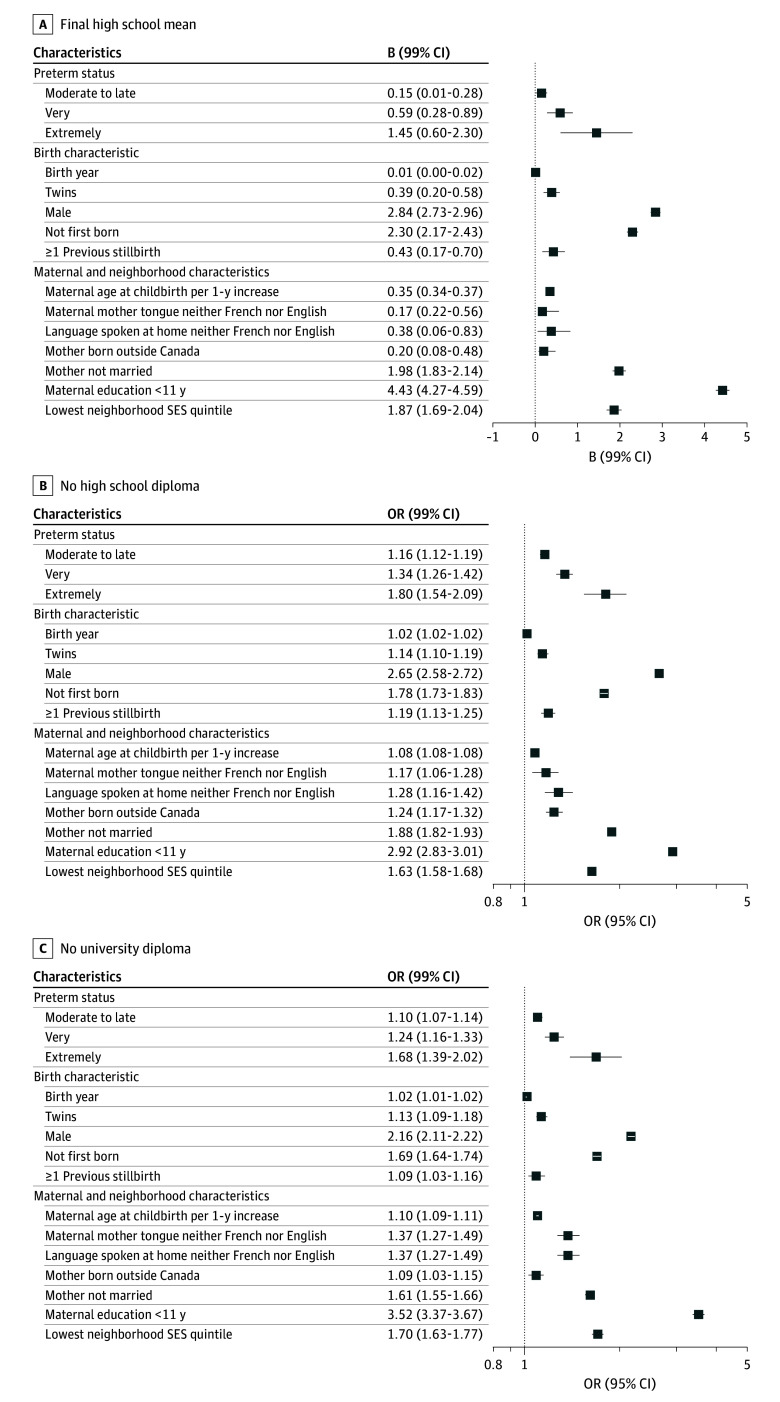
Paneled Forest Plot Showing Associations Between Preterm Status and Sociodemographic Factors and Educational Outcomes OR indicates odds ratio; SES, socioeconomic status.

#### High School Graduation

In the minimally unadjusted model, individuals born extremely preterm were at highest odds of not obtaining a high school diploma by age 22 years (OR, 1.88 [99% CI, 1.66-2.12]), followed by very preterm (OR, 1.43 [99% CI, 1.36-1.51]) and moderate-to-late preterm (OR, 1.22 [99% CI, 1.20-1.25]) (Table 2). After full adjustment, these associations were slightly attenuated. However, the effect size of the association with not obtaining a high school diploma was more important with factors other than preterm birth, notably low maternal education (OR, 2.92 [99% CI, 2.83-3.01]), male sex (OR 2.65 [99% CI, 2.58-2.72]), mother not married (OR, 1.88 [99% CI, 1.82-1.93]), low neighborhood SES (OR, 1.63 [99% CI, 1.58-1.68]), mother born outside Canada (OR, 1.24 [99% CI, 1.17-1.32]) and not being the first-born (OR, 1.78 [99% CI, 1.73-1.83]).

#### University Degree

Similarly to high school graduation, in the unadjusted model, individuals born extremely preterm were at highest odds of not obtaining a university degree (OR, 1.64 [99% CI, 1.40-1.92]), followed by very preterm (OR, 1.31 [99% CI, 1.23-1.39]) and moderate-to-late preterm (OR, 1.15 [99% CI, 1.12-1.18]). Once again after adjustment, factors other than preterm birth had higher effect sizes for the association with not obtaining a university degree, notably low maternal education (OR, 3.52 [99% CI, 3.37-3.67]), male sex (OR, 2.16 [99% CI, 2.11-2.22]), low neighborhood SES (OR, 1.70 [99% CI, 1.63-1.77]), not being first-born (OR, 1.69 [99% CI, 1.64-1.74]), and mother not married (OR, 1.61 [99% CI, 1.55-1.66]).

## Discussion

In this large, population-based, case-control study of educational outcomes in preterm and matched full-term births in Quebec, Canada, we found that although final high school average grades were similar, individuals born preterm faced greater challenges in graduating from high school or obtaining a university degree in a dose-response pattern with decreasing gestational age at birth. However, several sociodemographic factors outweighed preterm birth status, which has important implications for public policy: low maternal education (eg, reduced cognitive stimulation and lower earnings^25^), low socioeconomic status (eg, family stress and limited access to quality education and resources^26^), and male sex (eg, higher levels of externalizing behaviors^27^) had higher effect sizes for the association with school performance than preterm birth.

In the third trimester of pregnancy, cortical brain volume quadruples.^28^ In preterm birth, this development occurs ex utero, potentially entailing disruption in brain maturation and alteration in neurodevelopment.^13^ Depending on gestational age at birth, lower levels of school readiness,^29^ lower grades in school,^7^ lower IQ,^30^ and lower rates of completing basic education can be observed.^11,17^ Indeed, we found that individuals born extremely preterm had a 26% greater chance, as compared with those born term, of not graduating high school by age 22 years.

Our observed educational attainment rates (high school graduation, university degree) align with overall trends for the Canadian population. According to the Canadian Labor Force Survey,^31^ in 1990, 19% of women and 23% of men had no high school diploma by age 34 years (respectively 7% and 10% in 2009). For university education, in 1990, 86% of women and 83% of men had no university degree by age 54 years (72% and 75% in 2009, respectively). This confirms the observed biological sex gap, highlighting that male individuals tend to have lower educational attainment than female individuals. Although we did not include interactions between preterm birth and sex, meta-analytic evidence from 2024 suggests such interactions are negligible.^32^ Likewise, the observed increase in preterm births in Quebec, from 4.6% for 1976 to 1980 to 6.1% for 1991 to 1995, aligns closely with national prevalence estimates for those periods, which were 5.8% and 7.1%, respectively,^33,34^ highlighting the growing public health concern of children born preterm. This time period also saw major improvements in neonatal care (eg, expansion of neonatal intensive care units; Canadian Neonatal Network founded in 1995)^35^ and education reforms (eg, subsidized childcare in 1997),^36^ which would contribute to shaping outcomes observed in our cohort.

Certainly, not graduating high school would entail a lack of university education and correspondingly lower income levels. In addition, not graduating high school reduces possibilities for employment, which significantly influences quality of life.^37^ Furthermore, effect sizes for the associations between educational attainment and preterm status appeared relatively small compared with other sociodemographic factors. As such, public policy aiming to increase equity in educational attainment could likely focus on socioeconomic status, sex-based disparities, and additional support to immigrants as well as to families of children born preterm. Preterm birth can be a source of stress for families, with unexpected initial setbacks, time off work for health care appointments, and so on.^38^ Effective strategies such as improving access to quality public daycare^39^ could be prioritized, alongside ongoing monitoring and support for children born preterm who experience educational difficulties.^7^

Previous studies suggested that challenges following preterm birth persisted in adulthood, including lower education levels, greater unemployment, lower earnings, and greater reliance on welfare.^6,11,17,30,40^ Although evidence on eventual catch-up remained mixed,^15,20^ our findings in the present study indicated that individuals born preterm who took standardized examinations obtained similar marks to those of their peers. We also found that individuals born preterm were less likely to have registered grades within the Ministry of Education database following a dose response pattern. As individuals born preterm, especially those born extremely preterm, have higher rates of neurodevelopmental challenges (eg, intellectual and learning disabilities, autism spectrum disorders), they often benefit from specialized schooling that does not include these standard exams.^41^ The current study’s results could suggest that for children born preterm who receive proper support from the early years up to adolescence and who remain in the standard educational track, educational performance would be comparable with that of their peers. Nevertheless, higher rates of neonatal morbidity and complications among preterm individuals contribute to variability in educational outcomes and access to the standard educational track. Furthermore, the lack of difference in final high school average reflects in part a selection bias, as those born preterm were more likely to not finish high school and obtain final grades (eTable 2 in Supplement 1).

### Strengths and Limitations

Our study featured a large, robust longitudinal study design, including a 20-year population-based birth cohort with the full spectrum of all preterm births in Quebec, Canada. In case-control methodology, preterm births were rigorously matched 1:2 to a term control group from the same birth cohort. Second, our sample included all the extremely preterm births, whereas in prior studies they were often underrepresented due to low incidence (approximately 0.1% of live births). Third, we linked multiple administrative databases, eliminating bias associated with self-reported measures. Fourth, confounding factors for adjusted analyses included intergenerational information. Fifth, we conducted a rare in-depth analysis using the Quebec Ministry of Education databases up to age 43 years. We analyzed both educational attainment (high school graduation, university degrees) and academic achievement (final high school average, an indicator beyond pass or fail), for a more comprehensive understanding of preterm status on educational outcomes.

Our study also had several limitations. First, the range of educational programs and trajectories was large and heterogeneous. For instance, in Quebec, there are postsecondary professional diplomas that confer certifications for various trades, arts, and technical programs, but that are not classified as university degrees. For generalizability to other countries and research, we measured associations with university degrees, but thereby likely underestimated educational attainment in participants who obtained other types of professional diplomas. Also, we did not analyze domain-specific underachievement (eg, math, reading), which may vary by preterm status, use of educational interventions (eg, remedial learning), and rates of neonatal morbidities and learning impairments. Second, we used years of education as proxy for maternal high school graduation. In exceptional circumstances, it is possible to skip a grade and finish grades 1 through 11 in fewer years. Third, we used neighborhood SES as proxy for socioeconomic level, but taxation records if available might have provided further insights. We investigated multiple indicators of disadvantage but their cumulative effect remains unexplored. Furthermore, data on First Nations families were unavailable. In-depth analysis of sex and gender disparities was beyond the scope of this study. Lastly, the Quebec and Canadian context of universal health care and education may not generalize to other countries.

## Conclusions

This longitudinal, birth cohort case-control study found that children born preterm were less likely to earn high school or university diplomas than their full-term peers, suggesting that preterm birth may entail enduring learning challenges into adulthood, with vulnerabilities increasing as a function of gestational age at birth. Our findings substantiate the need for long-term follow-up of individuals born preterm, in both health care and education, particularly for individuals born extremely preterm. Associations of educational attainment (high school graduation, university degree) with preterm birth were minor compared to associations with socioeconomic status, male sex, and migration-related factors. Public policy therefore needs to prioritize issues of social inequity, plus provide additional support for families of children born preterm (eg, psychological counseling, physio- and occupational therapy, speech therapy and tutoring are not always covered by universal health care).

## Notes

Supplement 1.
eMethods.
**eTable 1.** Preterm Birth Rates in Quebec and Canada by Birth Year **eTable 2.** Missing Data in No High School Diploma and Final Average Grades in High School by Prematurity Status

Supplement 2.
Data Sharing Statement
