# Supplementary material for: Long-Term Educational Outcomes of Individuals Born Preterm
Source: JAMA Netw Open. 2025 Oct 1;8(10):e2534918. doi: 10.1001/jamanetworkopen.2025.34918 (PMC12489672; doi:10.1001/jamanetworkopen.2025.34918)
Supplement: Supplement 1. — eMethods. eTable 1. Preterm Birth Rates in Quebec and Canada by Birth Year eTable 2. Missing Data in No High School Diploma and Final Average Grades in High School by Prematurity Status [file jamanetwopen-e2534918-s001.pdf]

Supplemental Online Content

Loose T, Collet O, Nuyt AM, et al. Long-term educational outcomes of individuals born preterm. *JAMA Netw Open.* 2025;8(10):e2534918.
doi:10.1001/jamanetworkopen.2025.34918

eMethods.

eTable 1. Preterm Birth Rates in Quebec and Canada by Birth Year

eTable 2. Missing Data in No High School Diploma and Final Average Grades in High School by Prematurity Status

This supplemental material has been provided by the authors to give readers additional information about their work.

© 2025 Loose T et al. *JAMA Network Open*.

eMethods.

Neighborhood socioeconomic status (SES). We used the Material and Social Deprivation Index from the Quebec Public Health Institute (*Institut national de santé publique du Québec*, INSPQ). For each neighborhood, the index consists of 2 aspects of social inequality regarding inhabitants aged > 15 years: (a) social deprivation, i.e. living alone (single; separated, divorced or widowed; single-parent families); and (b) material deprivation, i.e. lack of material resources (low education; insecure job situation; insufficient income). Expressed in quintiles and based on the 3 first digits of the postal code, the index is provided every census year.[34] We obtained a residential history for each participant. Neighborhood SES was defined as the deprivation index at participant age 12, i.e. at high school entry (Grade 7) for most Quebec students. For participants born in 1976-1977, we used age 13, as the index was only available starting in 1989. We analyzed the most disadvantaged SES quintile as compared to others.

Maternal education level. As maternal education level was not available per se, we used years of schooling as cut-off for comparison. In Quebec, it typically takes 11 years, not including kindergarten (i.e. Grades 1-11), to graduate.

© 2025 Loose T et al. *JAMA Network Open*.

eTable 1. Preterm birth rates in Quebec and Canada by birth year

	Birth in Quebec[1]	Pre-term (<37 weeks) rates in our sample	Pre-terms (<37 weeks) rates in Canada[2]
Birth year, N (%)			
1976-1980	488,881	22,265 (4.55%)	5.76%
1981-1985	447,144	22,605 (5.06%)	5.92%
1986-1990	444,301	25,040 (5.64%)	6.12%
1991-1995	463,399	28,335 (6.11%)	7.08%

[1]Source: https://statistique.quebec.ca/fr/produit/tableau/naissances-et-taux-de-natalite-quebec

[2]Source: https://www150.statcan.gc.ca/n1/daily-quotidien/240925/cg-c001-eng.htm

© 2025 Loose T et al. *JAMA Network Open*.

eTable 2. Missing data in no high school diploma and final average grades in high school by prematurity status

	N valid		
	Final high school average	No high school diploma	Proportion
Term (37-42 wk)	161180	199575	0.81
Moderate to late preterm (32-36 wk)	63960	83105	0.77
Very Preterm (28-32 wk)	9645	13225	0.73
Extreme preterm (<28 wk)	1240	1915	0.65

© 2025 Loose T et al. *JAMA Network Open*.
