# Supplementary material for: Long-Term Educational Outcomes of Individuals Born Preterm
Source: JAMA Netw Open. 2025 Oct 1;8(10):e2534918. doi: 10.1001/jamanetworkopen.2025.34918 (PMC12489672; doi:10.1001/jamanetworkopen.2025.34918)
Supplement: Supplement 2. — Data Sharing Statement [file jamanetwopen-e2534918-s002.pdf]

Data Sharing Statement

Loose. Long-Term Educational Outcomes of Individuals Born Preterm. *JAMA Netw Open*. Published October 01, 2025. doi:10.1001/jamanetworkopen.2025.34918

Data
Data available: No
